# Proceedings of the 2022 “Lifestyle Intervention for Epilepsy (LIFE)” symposium hosted by Cleveland Clinic

**DOI:** 10.1002/epi4.13037

**Published:** 2024-08-23

**Authors:** Elizabeth Spurgeon, Robert Saper, Andreas Alexopoulos, Jane B. Allendorfer, Judith Bar, Jessica Caldwell, Mackenzie Cervenka, Sandra Darling, Stephen Dombrowski, Lisa Gallagher, Sara Lazar, Erik Modlo, Jim Perko, Martha Sajatovic, Bikat Tilahun, Nandan Yardi, Imad Najm

**Affiliations:** ^1^ Cleveland Clinic, Epilepsy Center Cleveland Ohio USA; ^2^ Department of Wellness and Preventive Medicine Cleveland Clinic Cleveland Ohio USA; ^3^ Department of Neurology University of Alabama at Birmingham Birmingham Alabama USA; ^4^ Cleveland Clinic Lou Ruvo, Center for Brain Health Las Vegas Nevada USA; ^5^ Epilepsy Center, Johns Hopkins University Baltimore Maryland USA; ^6^ Arts and Medicine Department Cleveland Clinic Cleveland Ohio USA; ^7^ Department of Psychiatry Massachusetts General Hospital Charlestown Massachusetts USA; ^8^ Cleveland Clinic, Center for Functional Medicine Cleveland Ohio USA; ^9^ Neurological and Behavioral Outcomes Center University Hospitals Cleveland Medical Center Cleveland Ohio USA; ^10^ Yardi Epilepsy Clinic Pune India

**Keywords:** cognitive behavioral therapy, music therapy, nonpharmacologic treatment, seizure control, yoga

## Abstract

Lifestyle interventions are strategies used to self‐manage medical conditions, such as epilepsy, and often complement traditional pharmacologic and surgical therapies. The need for integrating evidence‐based lifestyle interventions into mainstream medicine for the treatment of epilepsy is evident given that despite the availability of a multitude of treatments with medications and surgical techniques, a significant proportion of patients have refractory seizures, and even those who are seizure‐free report significant adverse effects with current treatments. Although the evidence base for complementary medicine is less robust than it is for traditional forms of medicine, the evidence to date suggests that several forms of complementary medicine including yoga, mindfulness meditation, cognitive behavioral therapy, diet and nutrition, exercise and memory rehabilitation, and music therapy may have important roles as adjuncts in the treatment armamentarium for epilepsy. These topics were discussed by a diverse group of medical providers and scientists at the “Lifestyle Intervention for Epilepsy (LIFE)” symposium hosted by Cleveland Clinic.

**Plain Language Summary:**

There are many people with epilepsy who continue to have seizures even though they are being treated with medication or brain surgery. Even after seizures stop, some may experience medication side effects. There is research to suggest that certain lifestyle changes, such as yoga, mindfulness, exercise, music therapy, and adjustments to diet, could help people with epilepsy, when used along with routine treatment. Experts discussed the latest research at the “Lifestyle Intervention for Epilepsy (LIFE)” symposium hosted by Cleveland Clinic.

## Key points

- This paper provides a detailed summary of the current evidence base for non‐pharmacologic lifestyle interventions in epilepsy and other select medical conditions, as presented at the 2022 educational symposium, “Lifestyle Intervention for Epilepsy (LIFE) hosted by Cleveland Clinic.
- Speakers presented valuable research‐based information in their area of expertise including topics such as yoga, mindfulness, cognitive behavioral therapy, exercise, individual and systems level influences, self‐management programs, diet, vitamins and supplements, and music therapy.

## 1 INTRODUCTION

A seminal 1993 survey of 1539 adults revealed that 34% of US adults used at least one alternative therapy in the previous year and that visits to providers of unconventional therapies numbered 425 million, with an out‐of‐pocket expenditure of $13.7 billion.^1^ These alternative therapies were mostly used in addition to mainstream treatments. Most users of alternative therapies, however, did not inform their doctor of such use. A similar rate of adoption of complementary health approaches was reported in the 2012 and 2017 National Health Interview Surveys conducted by the US Centers for Disease Control.

Deregulation of the dietary supplement industry in 1994 led to manufacturers making extravagant health claims without evidence to support them and limited the ability of health professionals and patients to meaningfully analyze the utility of these products. Angell and Kassirer in 1998 called for scientific testing of alternative medicine “no less rigorous than that required for conventional treatment”.^2^

In 1998, Congress established the National Center for Complementary Alternative Medicine (NCCAM) as part of the National Institutes of Health, with the ability to issue grants for rigorous research, unleashing an explosion of academic activity in alternative medicine. In 2014, NCCAM changed its name to the National Center for Complementary and Integrative Health, reflecting a shift in thought from products as alternatives to mainstream medicine to the integration of mainstream medicine with evidence‐based complementary therapies.

The Academic Consortium for Integrative Medicine & Health was founded in 1999. Its vision was a transformed healthcare system promoting integrative medicine and health for all, reaffirming the importance of the relationship between practitioner and patient and focusing on the whole person. A core value is the advance of integrative medicine and health that is informed by evidence and makes use of all appropriate therapeutic and lifestyle approaches, healthcare professions, and disciplines.

Despite the availability of more than two dozen medicines approved for the treatment of seizures in patients with epilepsy, a significant proportion of patients continue to experience seizures. In addition, patients with epilepsy often have difficult‐to‐address psychosocial implications such as loss of employment and independence, inability to drive, divorce, and psychiatric comorbidities, including depression, anxiety, post‐traumatic stress disorder, and psychosis. For them, lifestyle interventions can serve as useful adjuncts to traditional therapy and may offer patients with various types of epilepsy an opportunity to participate in their own care and the management of their disease.

On September 30–October 2, 2022, Cleveland Clinic hosted an educational activity “Lifestyle Intervention for Epilepsy (LIFE)” during which the evidence base for nonpharmacologic lifestyle interventions in select medical conditions and the current knowledge gaps of such interventions in epilepsy and its comorbidities were discussed. Not only were these proceedings in line with the mission priorities of NCCAM and the Academic Consortium for Integrative Medicine & Health but also with the National Institute of Health's Epilepsy Benchmark to “limit or prevent adverse consequence of seizures and their treatment across the lifespan”.^3^

The following sections of this report offer an in‐depth review of the literature: *yoga* (defining yoga, neurophysiology, and its effects on cognition, quality of life, and epilepsy), *cognitive behavioral therapy (CBT) and mindfulness training* (their effects on psychiatric comorbidities and seizure frequency, and review of neural mechanisms underlying these techniques), *physical activity and exercise in epilepsy* (review of data on the effects on seizure control, mental health, and cognition in epilepsy), *individual and systems level components influence on lifestyle change* (health literacy, motivation, self‐efficacy, and mental health), *self‐management programs in epilepsy, diet and memory loss* (nutritional impacts on cognition and neurodegenerative disease, ketogenic and low glycemic diets in epilepsy, and vitamins and supplements in epilepsy), and *music therapy* (review of the Mozart Effect in epilepsy, and further characterizing music therapy as an intervention).

## 2 YOGA

Yoga is a physical‐mental‐spiritual system of leading one's life. Beyond its physical aspects, yoga is a comprehensive and holistic practice, integrating both mental and spiritual dimensions. It involves a diverse range of mind–body practices such as meditation and relaxation techniques (dhyana), breathing practices (pranayama), physical postures (asana), and philosophical principles (yamas and niyamas). The poses, often simple to perform, in conjunction with conscious modulated breathing lead to a meditative state. There is a wide range of yoga styles or “schools” practiced which vary based on factors including pace, static vs. fluid postures, level of exertion, and relative contributions of different yoga components (e.g., postures, breathing, meditation). The specific style of yoga used should be taken into consideration when reviewing literature and its applications in this field. Yoga therapy offers a comprehensive yoga practice that includes a combination of these techniques, with the goal of reducing stress and improving physical, mental, and emotional health. The mindfulness and control of breathing is a key factor in most forms of Yoga. This practice is based on the most current medical and scientific findings and best clinical practice. In fact, yoga therapists receive additional training compared to yoga instructors and obtain certification through the International Association of Yoga Therapists.

The most promising study to date suggesting effectiveness of yoga for epilepsy was conducted by Kaur and colleagues in India.^4^ They conducted a 6‐month RCT of 160 adults with epilepsy, ages 18–60 years. They were randomized to 3 months of a semi‐standardized yoga therapy protocol consisting of warm‐up postures, breathing exercises, meditation, and relaxation with psychoeducation versus a sham yoga therapy without breathing, meditation, and relaxation, but with the psychoeducation for 3 months. The yoga group had higher odds of reaching greater than 50% seizure reduction (OR 4.11 95% CI [1.34, 14.69], *p* = 0.01) and complete seizure remission (OR 7.4 95% CI [1.75, 55.89], *p* = 0.005) compared to the control group. The yoga group also had statistically significant differences in felt stigma, anxiety, cognitive impairment.

The effects of yoga on neurophysiology have been studied. A predominant alpha spectral band is observed when the yoga practitioner is in a relaxed state practicing yogic high‐frequency breathing kapalabhati. Activation of the perirolandic region is caused by stimulation of visceral and splanchnic receptors through the vagus nerve. The breathing techniques practiced in yoga change intrathoracic and intra‐abdominal pressures in varying degrees. Parasympathetic discharge is increased, as studied in pranayama breathing exercises and Brahmakumaris Raja yoga meditation, while sympathetic discharge is reduced during the practice of yoga. On positron emission tomography (PET) scan, characteristic patterns of neural activity are observed with the practice of yoga meditation and indicate changes in cerebral glucose metabolism.^5^

In a pilot study, a 27% increase in brain gamma‐aminobutyric acid (GABA) levels was found in healthy volunteers after a 60‐min yoga (Asana) session versus no change in GABA levels in a comparator group after a 60‐min reading session.^6^ Two randomized controlled studies of yoga, one in healthy patients (*n* = 19) and one in individuals with major depressive disorder (*n* = 30), both found associations of yoga, increased GABA and improved mood.^7^, ^8^ Results from these studies suggest that yoga likely increases GABA levels. Whether this may be implicated in effectiveness of yoga in reducing seizure frequency, given the use of GABAergic or GABA‐targeted anti‐seizure medication, requires additional research.

Kriya yoga is a breathing and self‐realization meditation technique during which alpha activity is increased markedly, reflecting a strong conscious awareness. Volitional control and awareness of breathing during yoga engage distinct but overlapping brain circuits. During attention to breathing, increased coherence in the anterior cingulate, premotor, insular, and hippocampal cortices is observed.^9^

Various randomized controlled studies have shown yoga to alleviate stress, induce mental and physical relaxation, and provide multiple health benefits to those practicing it regularly. Continued practice of meditation was shown to synchronize and slow EEG activity and improve the clinical picture in individuals with drug‐resistant epilepsies.^10^ Improvements in spatial task capability, motor coordination, cognition, and mood have also been shown following 12 weeks of a yoga intervention.^11^

Quality of life benefits in people with drug‐resistant epilepsy were realized with group practice of asanas for 20 min daily. With 12 months of follow‐up, reductions in stress and apprehension were observed with subsequent reduction in the frequency of seizures. EEG and brain topography showed favorable results in 70% of patients.^12^

A Cochrane review of yoga as a complementary treatment, which included two unblinded randomized controlled trials of 50 adults with refractory epilepsy, showed significant improvement in quality of life with yoga according to the Satisfaction With Life Scale.^13^

**FIGURE 1:**
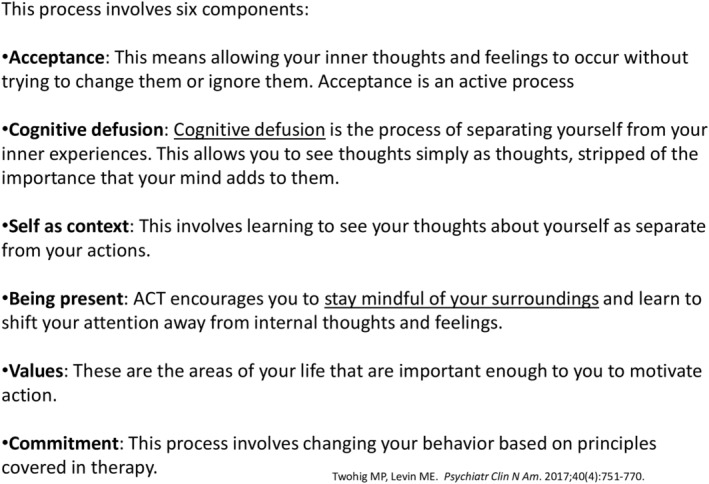
High‐level overview of the six components of acceptance and commitment therapy.^14^ Slide provided by Dr. Nandan Yardi as presented at the LIFE symposium.

The benefits of yoga may be comparable to a newer form of therapy called Acceptance and Commitment Therapy (ACT) (Figure 1). As detailed by its founder, Steven C. Hayes, PhD, ACT is developed based on the premise that suffering is a normal experience and that attempting to eliminate negative thoughts and feelings could lead to increased distress.^15^ Although it is not a fixed process, and instead highly individualized, the general aim of the acceptance phase consists of first recognizing unpleasant thoughts and feelings and the subsequent attempt at avoiding these experiences, and then learning how to instead embrace these challenging thoughts and strategies to achieve nonjudgmental awareness, through thought processes also known as cognitive defusion techniques. The goal of the commitment phase is then to apply this knowledge in accordance with one's values and goals. In one trial, ACT showed comparable therapeutic effects to yoga on measures of epilepsy, such as freedom from seizures at 1 year and reductions in seizure frequency, compared with baseline.^12^ This randomized single‐center trial of ACT offered as individual and group sessions was compared with yoga in subjects with drug‐refractory seizures and showed a ≥50% reduction in seizure frequency in nine of 10 participants in the ACT group and seven of eight in the yoga group (OR, 0.78, NS). At 12 months, six of 10 in the ACT group and four of eight in the yoga group had ≥50% reduction in seizure, suggesting promise for both interventions (OR, 0.67; 95% CI, 0.10–4.35). Freedom from seizures was achieved in five of 10 in the ACT group versus four of eight in the yoga group (OR, 1.00, 95% CI, 0.16–6.42). Although this data is promising, this study, the Cochrane review concluded that there was “low” quality evidence to support an effect on seizure control, due to lack of blinding and wide confidence intervals, and that further trials are needed.^13^

## 3 COGNITIVE BEHAVIORAL THERAPY (CBT) AND MINDFULNESS TRAINING

Psychiatric comorbidities are present in 25% to 50% of patients with epilepsy.^16^, ^17^ Epilepsy can trigger psychiatric disturbances such as depression and anxiety related to seizure‐related stressors, medication side effects, and altered brain activity in areas controlling mood regulation.^18^, ^19^ Patients with epilepsy can also develop PTSD.^20^ Psychiatric symptoms, in turn, can affect the patients' ability to manage their epilepsy by disrupting health routines such as sleep and medication adherence.^21^ This bidirectional relationship between psychiatric symptoms and seizures means evidence‐based behavioral interventions that ease psychiatric symptoms should be incorporated with traditional epilepsy treatment.

Cognitive behavioral therapy (CBT) is a well‐established psychotherapy approach considered effective in the management of chronic health conditions including epilepsy.^22^, ^23^ CBT addresses maladaptive beliefs and thoughts contributing to coping difficulties with adverse life events such as illness. In addition to the event itself, the interpretation of the event can interfere with healthy adjustment. The CBT intervention is low cost without serious side effects, can improve patients' engagement with their care, and is easily incorporated into the management of seizure disorders.

The goal of CBT is to improve coping with psychiatric symptoms, leading to illness acceptance, and to instill a sense of hope and empowerment, promote self‐care (e.g., sleep, exercise, diet), and increase social support. In CBT, both cognitive and behavioral interventions are used actively. The cognitive work requires modifying unhelpful thought patterns. These unhelpful thoughts are replaced with new, more productive alternative thoughts. In the following example, patients can be ambivalent to take medications daily and may tell themselves, “I am too young to take medications every day. I don't want to take my medications.” In CBT, they will be guided to identify adaptive thoughts such as “My medications give me the freedom to be active; Not taking them is worse than taking them; My friends have other challenges too.” CBT utilizes various techniques such as thought records and cognitive restructuring to empower patients to identify such unhelpful thinking habits and replace them with healthier ones.

CBT also utilizes behavioral interventions such as relaxation training and behavioral activation to increase socialization and engagement with pleasant activities. Unlike traditional talk therapy, CBT requires patients to take an active role in their treatment with required homework and assignments to be practiced outside of the counseling sessions. These strategies align with the ultimate goal of improving epilepsy outcomes in mood, function, quality of life, and seizure control.

Time‐limited CBT approaches have been successfully integrated in the treatment of medical conditions such as epilepsy.^24^ CBT can be delivered in individual or group therapy led by a mental health professional and involves skills training and assigned home practice. Integrating CBT with the specific aspects of the illness and modifying those illness‐related stressors and impairments showed promising outcomes.^25^

The Reiter/Andrews model, initially developed for the management of epileptic seizures, applied CBT techniques to treat drug‐refractory epilepsy and related psychological symptoms.^26^ Outcomes data of observational studies was promising for improving seizure burden; however, the model has not been tested within a randomized controlled trial (RCT) for the treatment of epileptic seizures. The Reiter/Andrews CBT model was redesigned in 2015 incorporating CBT and other seizure‐specific interventions and was renamed neurobehavioral therapy (NBT). NBT was modified to be used in the treatment of both epilepsy and psychogenic nonepileptic seizures (PNES) and accompanied by a treatment manual. The NBT manual has been tested for the treatment of PNES with an RCT that showed evidence that it improved seizure burden, mood, and quality of life.^25^ Most recently, NBT has been shown to improve cognitive function in PNES.^27^

**FIGURE 2:**
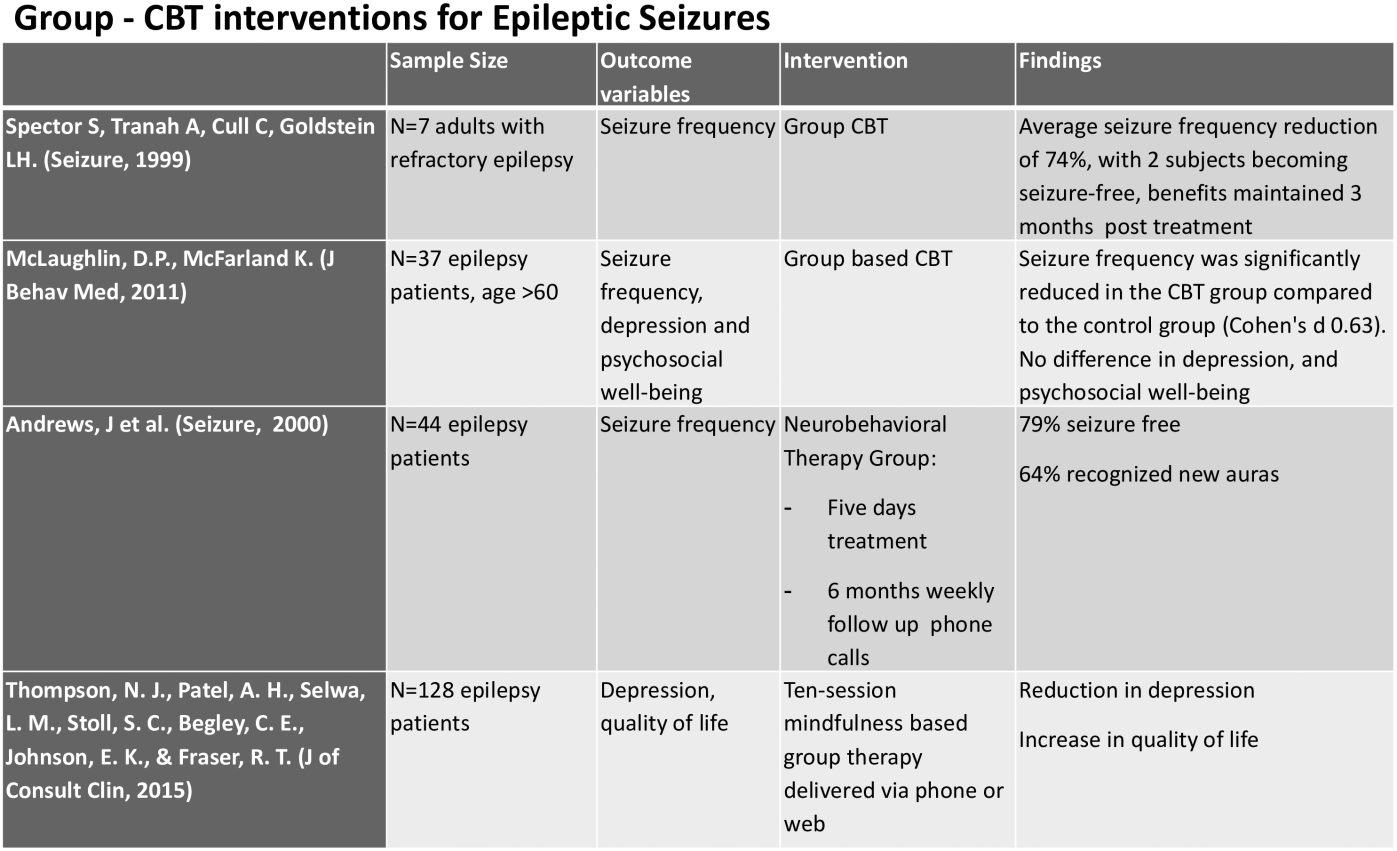
Cognitive behavioral therapy interventions for epileptic seizures. Slide provided by Dr. Bikat Tilahun as presented at the LIFE symposium.

A meta‐analysis review of several studies including RCTs found CBT therapy to be effective for reducing psychiatric symptoms and some support for reducing epileptic seizure frequency (Figure 2).^23^, ^28^ Some of these data, however, were limited by small sample sizes, partial randomization and control, and limited blinding, thus were rated on the PEDro‐P scale as moderate quality studies. More data therefore are needed to replicate these findings.

CBT encompasses traditional and newer CBT‐aligned psychotherapy practices such as mindfulness‐based therapies. Mindfulness is a state of calmness and stability as well as a trait characterized by the tendency to observe present‐moment perceptions, emotions, thoughts, and actions without judgment. Although practiced differently from traditional CBT, mindfulness‐based interventions are found effective in promoting radical acceptance and positive adjustment in life.^29^ In mindfulness training, individuals practice being present‐minded, nonjudgmental, and self‐aware. Mindfulness‐based interventions enable individuals to focus less on judging one's unfortunate life circumstances and more on finding purpose and meaning in life despite the difficulties. Mindfulness practice, therefore, can modulate the stress response to epilepsy by creating a sense of acceptance and promoting healthy adjustment.^30^

Mindfulness‐based psychotherapy approaches, which are designed to induce a state of mindfulness, are promising models for self‐management in epilepsy.^25^ A review of three RCTs showed that mindfulness‐based interventions are effective in alleviating the psychiatric comorbidities in epilepsy and can improve quality of life.^31^

Evidence is emerging for improvement in quality of life and mental health comorbidities of epilepsy with MBSR. Compared with social support, mindfulness was associated with greater reductions in symptoms of depression and anxiety and seizure frequency in an assessor‐blinded study of adults with drug‐resistant epilepsy. ACT was also found effective within a randomized controlled trial for reducing seizure burden.^32^ However, due to the poor quality of the studies and limited number of trials, no strong conclusions could be made.

Group‐based CBT interventions in patients with seizure disorders were effectively used to reduce seizure frequency compared with randomized controls, improve the proportion of patients who were free from seizures, reduce the severity of depression, improve quality of life measures, and reduce the number of negative health events (e.g., emergency department visits).^33^, ^34^, ^35^ In addition, group‐delivered mindfulness‐based CBT in adults with epilepsy and comorbid depression resulted in an improvement in depressive symptoms, knowledge/skills and life satisfaction versus controls on a wait list for the program.^35^

### 3.1 Neural mechanisms underlying the beneficial effects of mindfulness training

**FIGURE 3:**
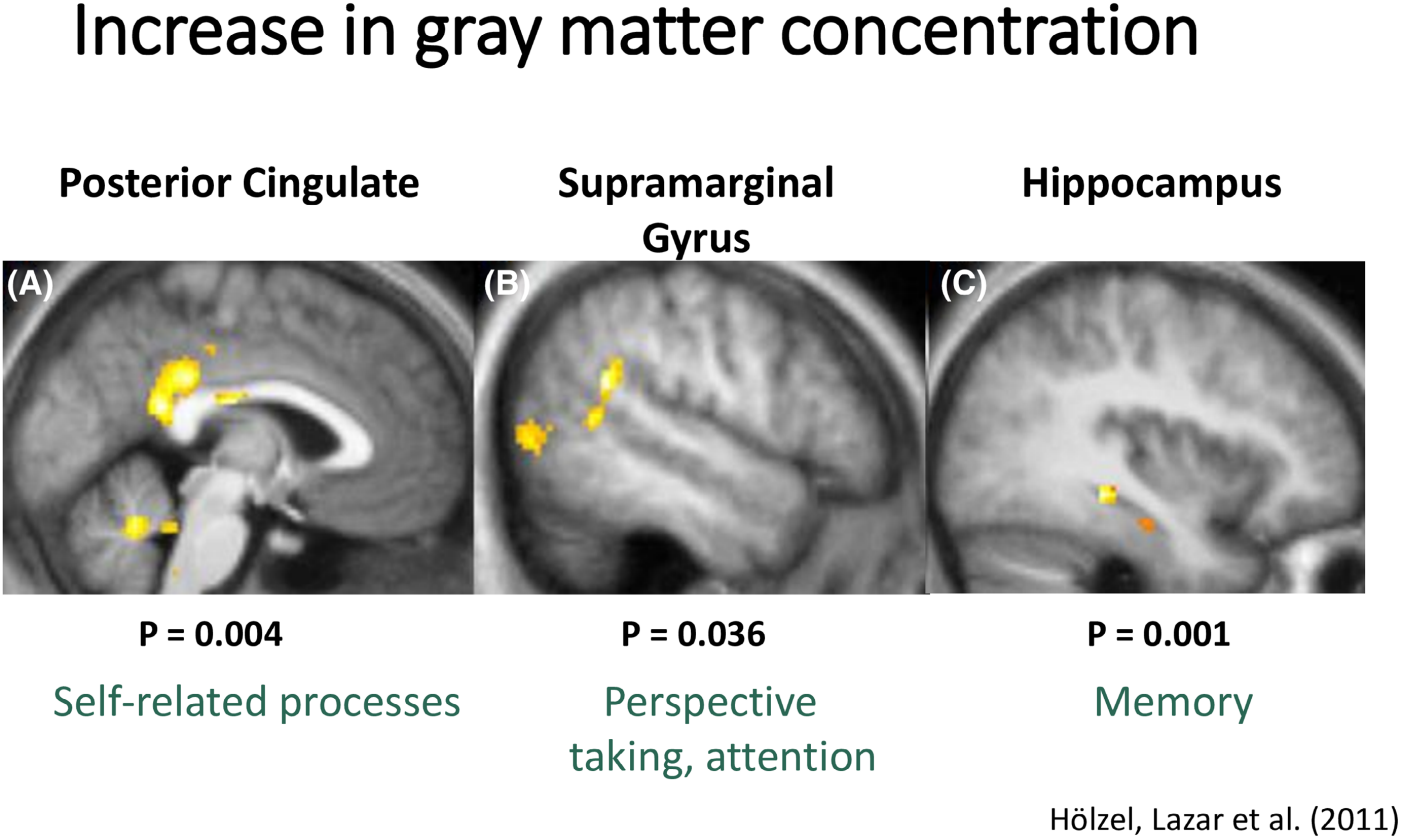
Increase in gray matter concentration in the (A) Posterior cingulate, (B) Sumpramarginal gyrus, and (C) Hippocampus, after Mindfulness‐Based Stress Reduction (MBSR) using voxel‐based morphometry.^36^ Slide provided by Dr. Sara Lazar as presented at the LIFE symposium.

Changes in gray matter have been observed with mindfulness training; specifically, an increase in gray matter concentration occurs with meditation (Figure 3).^36^ The change in amygdala gray matter correlates with a change in perceived stress.^37^ With MBSR increased gray matter in the hippocampus inversely correlates with hippocampal‐lateral occipital (visual) cortex functional connectivity while viewing fear‐inducing images, and the reduction in connectivity correlates with a reduction in anxiety.^38^ Conversely, connectivity between the hippocampus and sensory cortex while viewing these images increases following MBSR. Finally, activity in the supramarginal gyrus, which helps direct attention either internally or externally, was also enhanced while viewing these images. Together, these data indicate that mindfulness training enhances body awareness and that this enhanced awareness is associated with lower anxiety.

## 4 PHYSICAL ACTIVITY AND EXERCISE IN EPILEPSY

Inactivity is common among people with epilepsy, irrespective of seizure control, leading to suboptimal fitness.^39^, ^40^ About half of people with epilepsy have cognitive deficits, especially in memory.^41^, ^42^

The World Health Organization defines exercise as a subcategory of physical activity in which the activity is planned, structured, repetitive, and purposeful. In healthy adults, exercise improves performance on tests of memory, attention, processing speed, and executive function, and in mid‐life, exercise reduces the risk of dementia and mild cognitive impairment.^43^

The potential mechanisms by which exercise benefit's cognitive function include an increase or preservation of brain volume, particularly in the hippocampus, and changes in connectivity (structural and functional).^44^ Exercise reduces the severity of cardiovascular and cerebrovascular risk factors associated with dementia and increases production of brain‐derived neurotrophic factors, compounds that increase neurogenesis and are important in learning and memory functions in animals and humans.^43^ In animal models of epilepsy, exercise has also been shown to benefit memory function and other cognitive functions, with exercise‐induced changes in hippocampal long‐term potentiation^45^ and hippocampal DNA methylation^46^ as potential mechanisms of action.

Exercise is considered safe in most people with epilepsy,^47^ as it does not alter the metabolism of antiseizure drugs, is not seizure inducing, and may reduce seizure frequency.^48^, ^49^, ^50^ Interictal epileptiform discharge, an EEG biomarker for epilepsy, has also been shown to decline during exercise and/or in the postexercise recovery period.^51^, ^52^, ^53^ A recent systematic review and meta‐analysis showed a trend of decreased seizure frequency with moderate exercise in people with epilepsy.^54^ In this review of the data, exercise activities included stationary cycling, resistance training (such as bicep curls, leg presses, etc.), walking or running on a treadmill, strength training with exercise equipment, supervised activities (basketball, soccer, dance, etc.), and home‐based aerobic and flexibility exercises. Intensity of exercise in the majority of these studies was determined by reaching at least 60% of their maximal heart rate, 60% peak VO_2max_ (maximum rate of oxygen consumption), or reaching moderate intensity level on the Rated Perceived Exercise Scale.^54^ It should be noted that there are variations in the field with regard to definitions for different levels of exercise intensity, although there is a consensus that intensity is an important factor in the beneficial effects of exercise.^55^ More definite conclusions from this systematic review could not be made due to limitations of the data which include heterogeneity in the design of the studies, low number of patients, and the diversity in exercise programs evaluated (e.g., variations in the modality, frequency, and duration used for exercise activities).

Exercise intervention can improve both physical and mental health in epilepsy. Studies of supervised exercise demonstrated improvements in physical state, physical strength, mental state, emotional well‐being, and psychosocial functioning^48^, ^50^, ^56^ in people with epilepsy. Studies of home‐based exercise programs showed improvements in physical fitness,^57^ as well as general health, quality of life, and psychosocial functioning.^58^ A recent meta‐analysis showed a significant effect of exercise on improving quality of life in people with epilepsy.^54^ Physically active people with epilepsy have significantly lower levels of depression than those who are less active.^59^ Thirty‐five weeks of home‐based exercise improved attention and executive function in children with benign epilepsy.^58^ Combined endurance and resistance exercise improved verbal learning, memory, and functional connectivity in adults with epilepsy.^56^ The change in verbal memory may be mediated by a change in hippocampus resting state functional connectivity. The first RCT of the effects of exercise on cognition in people with epilepsy showed improvements in executive function, verbal fluency, and overall cognition in the intervention group.^60^ Although cognitive benefits of exercise in people with epilepsy are promising, further studies are necessary to confirm these effects.^61^

## 5 INDIVIDUAL AND SYSTEMS LEVEL COMPONENTS INFLUENCE ON LIFESTYLE CHANGES

Certain individual and system‐level factors may enable or restrict an individual from effecting lifestyle change. In order to best support change in patients with epilepsy, it is key to take a whole‐person approach to understanding these resilience and risk factors. Individual factors that may have an impact on one's ability to make and maintain change include health literacy, motivation, self‐efficacy, and mental health.

Health literacy is defined by the National Institutes of Health as the ability of an individual to find, understand, and use information and services to inform health‐related decisions.^62^ Importantly, NIH further emphasizes that health literacy does not solely rest in the hands of an individual but also comprises organizational responsibilities. Organizational health literacy is the degree to which organizations equitably enable individuals to perform these same functions.

A second factor in making and maintaining change is motivation—the desire to act to reach a goal and the ability to initiate, guide, and maintain goal‐oriented behavior. High motivation predicts adherence to lifestyle change in chronic disease, whereas low trust in providers predicts nonadherence, specifically in historically marginalized communities such as African Americans.^63^, ^64^ A perceived benefit to a change in behavior is essential to following through on the act of change.

Self‐efficacy is the belief in the ability to carry out behaviors that will lead to specific outcomes and is a common predictor of lapse/relapse in diet and exercise behavior.^65^ Specifically, in people with epilepsy, it has been found that the potential benefit to improve physical and mental health was a common motivating factor in maintaining exercise.^66^ In other words, knowledge of health conditions and motivation to change are not necessarily sufficient for change to occur if individuals doubt their own ability to make and sustain changes.

Mental health conditions, such as anxiety, depression, and life stressors, can have a significant impact on one's motivation, self‐efficacy, and thus participation in lifestyle changes. It has been observed that clinically diagnosed depression and high life stress can lead to treatment nonadherence in chronic conditions.^67^

Moving beyond individual factors, systems level characteristics also have an impact on individuals' ability to change successfully. These factors include family and close friends, neighbors and local resources, and regional and national settings, all of which can serve to support or hinder individuals who desire to make a change. In fact, in regard to physical activity, many people with epilepsy report that they have been “advised to avoid most types of exercise by a physician” or have been “discouraged from exercising by family and/or friends”.^59^ Social determinants of health, including access to medication, health services, fresh food, money, education, and transportation also influence the ability of individuals with epilepsy to make lifestyle changes.^68^ Without considering and addressing these larger systems factors, patients–and especially historically marginalized individuals–may be left with a recommendation, but without a clear route to making and sustaining the recommended change.

A personalized approach to change emphasizing both social and psychological risk and resilience can enhance the likelihood of success.^69^ Discovering the best strategies to support change in epilepsy is an area of research need. One possible tool is motivational interviewing techniques, which can promote adherence to behavioral changes and improve outcomes in clinical trials; however, more evidence is needed to assess its impact in clinical settings.^70^ Even without strict adherence to a motivational interviewing model, techniques rooted in motivational interviewing may be helpful in allowing treating providers to understand whether a patient is ready to enact change—for example, according to the stages of change model (i.e., precontemplation, contemplation, preparation, action, maintenance, relapse).^71^ Tools from motivational interviewing may also provide clinicians with mnemonics for assessing individual and systems level factors that may impact the ability to change (e.g., DARN acronym: D, desire; A, ability; R, reasons; and N, need).^72^

## 6 SELF‐MANAGEMENT PROGRAMS IN EPILEPSY

Another important lifestyle strategy consists of evidence‐based epilepsy self‐management programs. These programs incorporate educational and coping strategies that occur between a patient with epilepsy, their families, and their provider. They address specific components such as treatment of seizures, tracking and managing seizures when they occur, avoiding triggers, and optimizing health through lifestyle changes. The Managing Epilepsy Well (MEW) Network, supported by the Centers for Disease Control and Prevention (CDC), conducts research on self‐management programs to ensure their evidence‐based quality and to promote utilization in practice. One example of self‐management programs is HOBSCOTCH (Home Based Self‐management and Cognitive Training Changes Lives), which consists of learning how cognition is affected in those with epilepsy, self‐awareness training, and learning tools to compensate and adapt during day‐to‐day life. Another program, Self‐management for people with epilepsy and a history of negative health events (SMART), has a broader and multicomponent focus. SMART has been demonstrated to reduce epilepsy‐related complications and improve quality of life and physical and mental health functioning.^73^ Other evidence‐based self‐management programs focus on addressing depression and mental illness, enhancing overall health, and improving communication to healthcare providers.^74^

## 7 DIET AND MEMORY LOSS

A healthy lifestyle that incorporates a diet with key nutrients that feed the brain may help prevent mild to moderate memory loss. Subjective cognitive impairment and mild cognitive impairment (MCI) are often preceded by a preclinical stage, sometimes by 20 years or more. About 10% to 15% of persons with MCI will progress to dementia every year. These early stages are considered modifiable. Early action to protect and support the brain through lifestyle intervention, such as diet, may reverse early impairment or at least delay its progression, in part by reducing the severity of vascular risk factors. Diets that have been studied for this purpose include the Mediterranean diet, a whole food plant‐based diet, DASH diet, and the MIND diet, which is a hybrid of DASH and Mediterranean diets. Unprocessed plant foods constitute the backbone of these diets, with little or no contribution from animal foods. The MIND diet contains a specific set of guidelines that are easy to follow, with an abundance of antioxidant‐containing complex carbohydrates (3 servings of whole grains per day, berries 2 times/week, dark leafy greens 6 times/week, 1 additional vegetable/day, and beans 3 times/week).

Persons with high adherence to the MIND diet had a 53% lower risk of developing Alzheimer's disease (AD) and those with moderate adherence had a 35% lower risk than those with low adherence.^75^ Both the DASH and Mediterranean diets were also associated with a lower risk of AD when compliance was high, but only individuals in the highest tertile of adherence to a DASH or Mediterranean diet show a reduced risk of AD compared with those in the lowest tertile. High intake of leafy greens (1–2 servings/day) by itself has been shown to slow cognitive decline.^76^ Evidence across multiple studies suggests that lutein‐containing greens may prevent cognitive decline.^77^

Hypertension causes damage to the brain vasculature, causing cognitive impairment and stroke. A diet high in nitrates, found in green leafy vegetables and beetroot, improved cerebral perfusion in the frontal lobes of older adults while lowering blood pressure.^78^

Herbs and spices such as turmeric, saffron, and rosemary are rich sources of antioxidants shown to improve memory and cognitive function.^79^ Cocoa is also rich in antioxidants and flavonols; drinking cocoa flavonols results in acute improvement in cognitive performance.^80^

The MIND diet also incorporates healthy fats, such as those from fish, extra virgin olive oil (which should be the primary oil used for cooking), and nuts (5 times/week). Walnuts, flaxseed oil, chia seeds, and fatty fish are sources of omega‐3 fatty acids, shown to reduce the risk of dementia.^81^

Adequate amounts of water are necessary daily to avoid dehydration, as mild dehydration can affect brain function.^82^ The exact amount of water needed depends on time spent doing physical activity and the amount of fruits and vegetables consumed. A low level of alcohol consumption appears to reduce the risks of AD and dementia.^83^ The odds of hippocampal atrophy are greatly increased in moderate and heavy alcohol drinkers compared with abstainers.^84^

### 7.1 Ketogenic and low glycemic diets in epilepsy

The observation that fasting produces a state of ketosis that can control seizures dates back several centuries. In the 1920s, fasting was considered an effective treatment for epilepsy based on case series. In 1930, Barborka published the results of a study of a ketogenic diet practiced for 1 to 42 months in 100 patients with epilepsy, with the finding that 56% of the patients derived benefit.^85^ A ketogenic diet re‐emerged in the 1990s primarily as a treatment for children with epilepsy.

**FIGURE 4:**
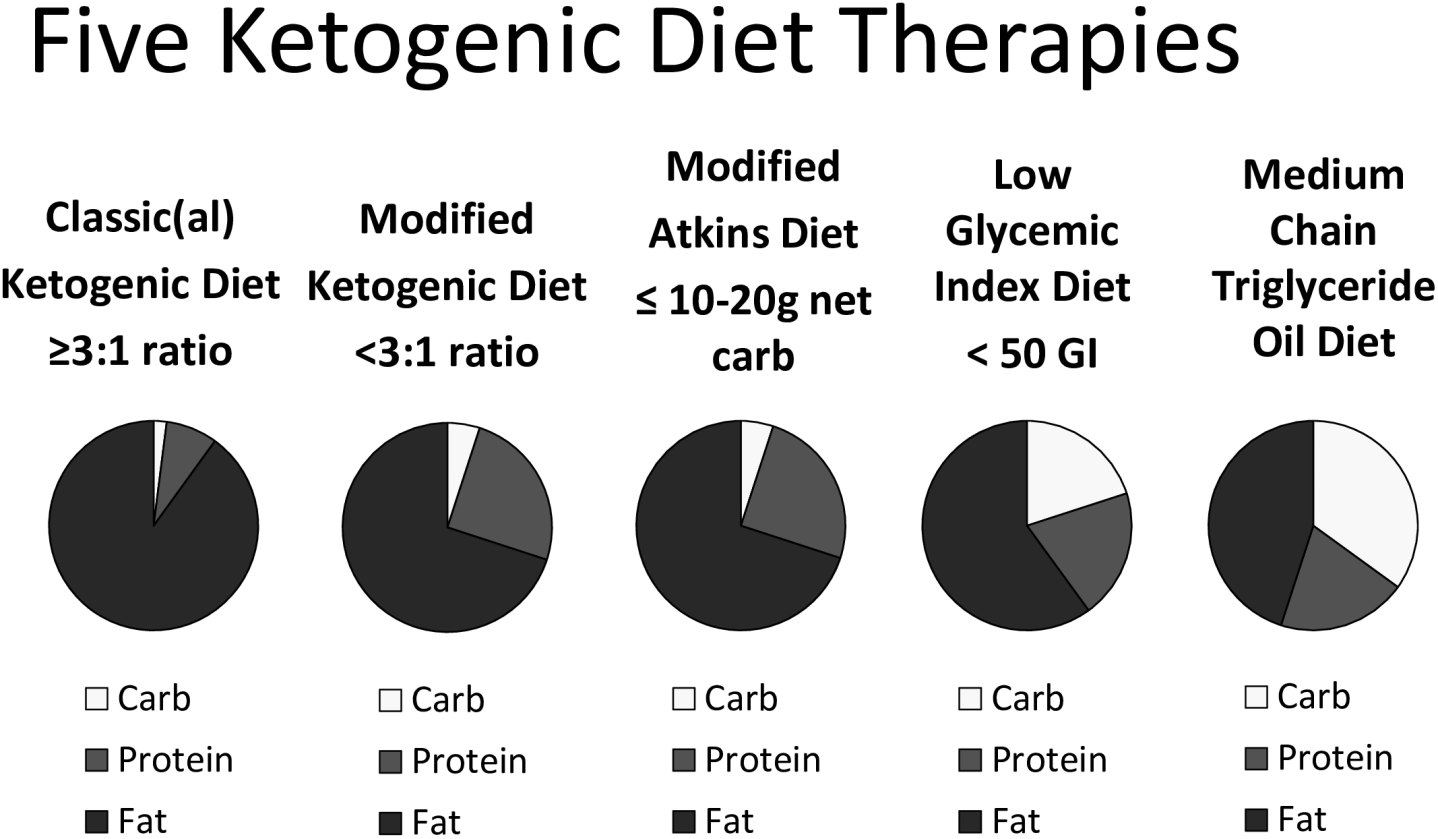
Ketogenic diet therapies. Slide provided by Dr. Mackenzie Cervenka as presented at the LIFE symposium. Carb, carbohydrate; GI, glycemic index.

Ketogenic diet therapies are classified as follows: classic ketogenic diet, modified ketogenic diet, modified Atkins diet, low glycemic index treatment, and medium chain triglyceride oil diet (Figure 4). Each has its own composition and selection of macronutrients including fat, protein, and carbohydrates to achieve ketosis.

The classic ketogenic diet derives about 90% of its calories from fat. It induces ketone body production through metabolism of fat. Nutritionally complete enteral formulas are available, typically with a 3:1 or 4:1 ratio of fat to carbohydrates plus protein combined in grams. Use of these diets for medical purposes may involve a hospital admission and fasting at onset.

The modified ketogenic diet contains a fat‐to‐carbohydrate ratio of approximately 1:1 or 2:1 with the same principles as the classic ketogenic diet. The medium chain triglyceride diet derives 30% to 60% of its calories from MCT oil (from coconut or palm kernel oil). Although a state of ketosis is reached quickly with this diet, gastrointestinal side effects have limited its popularity. The modified Atkins diet employs 10–20 g net daily carbohydrate intake (subtracting fiber), depending on a person's age. Constipation is a frequent side effect of these diets. The low glycemic index treatment (LGIT) emphasizes complex carbohydrates over simple sugars. Total carbohydrate intake is 40 to 60 g/day of foods with glycemic index ≤50. Ketosis is not the primary goal of LGIT but rather the foods with a low glycemic index slow digestion/absorption of a carbohydrate food, leading to a gradual rather than rapid rise in glucose and insulin. The emphasis, therefore, is on stability of blood glucose and insulin levels.

Ketogenic and low glycemic index diets work to prevent seizures in multiple ways. One proposed mechanism is through anti‐inflammatory properties, as rapid glucose fluctuations promote inflammation, and ketone bodies themselves reduce the secretion of proinflammatory chemokines and cytokines through interruption of the NLRP3 inflammation pathway.^86^

The average response rate for a ketogenic diet in the treatment of children with epilepsy is approximately 50%, when defined as >50% reduction in seizure frequency. However, response rates can exceed 70% for several epilepsy syndromes and conditions, such as tuberous sclerosis complex, Rett syndrome, Lennox–Gastaut syndrome, glucose transporter type 1 deficiency syndrome, genetic generalized epilepsies, and focal epilepsies due to underlying migrational disorders.^87^ Head‐to‐head trials of ketogenic and low glycemic trials with antiseizure medications are needed to determine which diet is most effective for specific seizure types.

A prospective randomized trial comparing a modified Atkins diet, LGIT, and the classic ketogenic diet as adjuncts to antiepileptic drug therapy in pediatric patients with refractory seizures demonstrated noninferiority of the modified Atkins diet and LGIT to the classic ketogenic diet on seizure frequency reduction over 24 weeks.^88^

In a prospective open‐label 6‐month study of 30 adults with an average seizure frequency of 10/week, 87% of patients enrolled remained on a modified Atkins diet for ≥1 months and 67% for ≥3 months. At both 1 month and 3 months, 47% had ≥50% reduction in seizure frequency, and at 6 months, 33% had a ≥50% reduction in seizure frequency. The effect of the diet was rapid (within 1 week). A potential explanation for the decreasing effect over time is the high rate of attrition: only about half of the participants remained on diet therapy at study completion.^89^

Although ketogenic diets have been used safely and effectively for longer than one century to treat people with epilepsy, they should only be used with medical and nutritional supervision to prevent adverse effects and to optimize therapeutic benefits. Without medical supervision, kidney stones, hyperlipidemia, and bone loss are some of the recognized long‐term risks.

Regardless of the specific diet type, cooking healthy meals involves skill and knowledge and may present limitations depending on time, budget, and access to resources. Culinary Medicine is a new, rapidly evolving evidence‐based field of medicine that combines the evidence‐based science of food, nutrition, and medicine with the joy and art of cooking.^90^, ^91^ It aims to increase culinary literacy and skills so that patients can execute their prescribed meal plans and find satisfaction in meal preparation, with a goal of achieving sustainability so they don't compromise the taste of their meals or their health.

### 7.2 Vitamins and supplements

Much of the research on the utility of vitamins and supplements for seizure prevention in patients with epilepsy suffers from poor‐quality studies with low sample sizes. The most convincing data lie with vitamin B6 supplementation in newborns and infants with vitamin B6 deficiency caused by autosomal recessive inheritance. Vitamin B6 is a water‐soluble vitamin that is vital to the development and maintenance of the central nervous system. Vitamin B6 (pyridoxine)‐dependent seizures most often present in the neonatal period until age 3 and cause intractable seizures.^92^

Pyridoxal 5′‐phosphate (PLP) is involved in the synthesis of multiple neurotransmitters, including GABA. A deficiency of cellular PLP can cause abnormal glutamic acid decarboxylase binding site affinity, leading to lower levels of GABA production.^93^ With vitamin B6 administration at dosages of 100–200 mg delivered intravenously, clinical seizures typically resolve within days. The maintenance dosage is variable (10–200 mg), with a target dosage of 25–50 mg/day. Upon cessation of B6 support, seizures can return in 48–72 h.

Significantly lower serum pyridoxine levels were found in adult (age range: 25–57 years) patients with poor control of their seizures compared with those with well‐controlled seizures in a single‐center cross‐sectional study of 30 patients with epilepsy (*p* < 0.03).^94^ A limitation of this study was the definition of poor control: “no provoked seizure event” in the preceding 3 months.

Magnesium has a role in the reduction of inflammation and upregulation of GABA synthesis in the brain. Magnesium intake <350 mg/day was associated with a significantly higher risk of epilepsy (*p* < 0.002) in middle‐aged men enrolled in the Kuopio Ischaemic Heart Disease Risk Study who were followed for >22 years,^95^ but no evidence to date supports magnesium supplementation as a treatment to reduce seizure frequency. Other vitamins/supplements for which low‐quality data suggest antiepileptic effectiveness but for which no recommendation can be made are taurine, vitamin E, vitamin D, vitamin B1, vitamin B9, polyunsaturated fatty acids, and zinc.

Studies of cannabinol for the treatment of epilepsy are becoming more common, with several ongoing. No reliable conclusions could be drawn about the efficacy of cannabinoids as a treatment for epilepsy, according to a 2014 Cochrane Review^96^ of four randomized placebo‐controlled studies of 200–300 mg of cannabinol that enrolled a total of 48 patients.

Cannabidiol has proven efficacy for specific epilepsy syndromes and has an indication for use in the treatment of seizures associated with Lennox–Gastaut syndrome, Dravet syndrome, or tuberous sclerosis complex (TSC) in patients 1 year of age and older. A 100 mg oral cannabinol solution administered for 14 weeks was found to reduce seizure frequency by 39% (*p* = 0.01) in a randomized, double‐blind, placebo‐controlled trial of 120 patients with Dravet Syndrome.^97^ In patients with Lennox–Gastaut Syndrome, 20 mg/kg oral cannabidol administered daily for 14 weeks reduced seizure frequency by 43% (*p* = 0.01) in a randomized, double‐blind, placebo‐controlled trial that enrolled 171 patients.^98^

## 8 MUSIC THERAPY

Music therapy is defined as clinical and evidence‐based use of music interventions to accomplish individualized goals, be it pain relief, enhancement of memory, or wellness promotion, within a therapeutic relationship by a credentialed professional who has completed an approved music therapy program.^99^ Engagement with the arts can help reduce stress, assist with coping, and improve body, mind, and spirit.^100^, ^101^

Although the research on music as a therapy for epilepsy is limited, the theoretical background for music in epilepsy is rich. Music is a form of neuromodulation. Sensory stimuli, when attended to, can have widespread effects within the brain. Exposure to music exploits reward systems that stabilize brain states that are healthier than other brain states. Because music is a stimulus that modulates brain activity, it can be used to break the pattern of epileptiform activity to a more synchronous type of activity in the cortex that typifies healthy brain dynamics.

In 1993, Rauscher et al. described the Mozart Effect, an enhancement of spatial reasoning skills after listening to 10 min of Mozart K.448, an organized, repetitive sonata for two pianos with relatively unpredictable rhythmic structure.^102^ It appears that Mozart's music provides the equivalent of patterned electrical stimulation in patients with epilepsy. Mozart K.448 has been shown to release mirror neurons and improve the activity of dopamine. In a study of 29 patients with seizures exposed to K.448, 80% had a reduction of epileptiform activity; a patient that was comatose with status epilepticus also had a reduction in ictal activity by about two thirds during the music.^103^ The Mozart K.448 effect was reproduced in patients with refractory focal epilepsy undergoing intracranial EEG recordings; the effect was absent with other musical stimuli.^104^ Meta‐analyses support the positive effect of K.448 on interictal discharge rates and seizure reduction,^105^ although most of the studies lacked another sound stimulus as a control and other limitations included low number of studies, small sample sizes, and heterogeneity of protocols.

A controlled crossover study in which individuals with epilepsy were randomized to 3 months of daily exposure to Mozart K.448 or a scrambled nonrhythmic piece that served as control confirmed the effect of Mozart K.448 on reducing seizure frequency that was absent during the control listening period.^106^ The authors proposed that the unpredictability in rhythmic structure in Mozart K.448 makes brain activity less predictable and less like seizure activity and that listening to Mozart K.448 reshapes brain activity by means of a 1/f resonance mechanism.

When working with any individual, considering their musical preferences is important, as research has found that a patient's preferred music will be the most effective music for them to use.^107^ Therefore, individuals should be encouraged to create their own playlists for various activities, such as exercising or cleaning, to represent different moods, and to assist with relaxation and sleep. Intentional listening is also an important aspect of using music as it has the ability to cause reactions throughout the body, stir emotions, and bring about memories.^108^ Since stress can cause an increase in the frequency of seizures, teaching patients a variety of stress management and relaxation techniques is also essential. Some of these techniques could include deep breathing, progressive muscle relaxation, relaxation response, and music‐assisted imagery.^109^, ^110^, ^111^, ^112^, ^113^ Music therapists are trained in how to incorporate these techniques into patients' treatment, as well as the utilization of specific music therapy interventions such as singing, instrument playing, improvisation, songwriting, and movement to music. Music makes movement more interesting, it provides a pleasant distraction, it energizes and motivates, and it increases the release of endorphins.^114^, ^115^, ^116^, ^117^ Although little research has been done yet specific to epilepsy, these music therapy interventions have been investigated and shown to be beneficial for a wide variety of conditions.

## 9 CONCLUSION

While the use of lifestyle interventions in medicine remains in its adolescent phase of growth, the acceptance of and evidence base for lifestyle and wellness in health care is growing. Reductions in seizure frequency and interictal discharge rates, improvements in quality‐of‐life measures and physical and mental health, and a reduction in the number of negative health events have been realized with some complementary medicine techniques in patients with epilepsy. Research that is rigorous and interdisciplinary using multiple methodologies is critical to map the way forward if lifestyle interventions are to be integrated into epilepsy care in mainstream healthcare settings.

## AUTHOR CONTRIBUTIONS

E.S. assembled the team of authors; wrote the first draft of the manuscript and finalized the manuscript. E.S., R.S., and I.N. participated as co‐directors of the LIFE symposium; provided content information; edited and revised the manuscript. A.A., J.B.A., J.B., J.C., S.D., S.D., L.G., E.M., and M.S. provided content information; edited and revised the manuscript. M.C. provided content information; edited and revised the manuscript; contributed presentation slide for Figure 4. S.L. provided content information; edited and revised the manuscript; contributed presentation slide for Figure 3. B.T. provided content information; edited and revised the manuscript; contributed presentation slide for Figure 2. N.Y. provided content information; edited and revised the manuscript; contributed presentation slide for Figure 1.

## CONFLICT OF INTEREST STATEMENT

Dr. Mackenzie Cervenka is a consultant for Nutricia North America/Danone, Nestle Health Science, University of Maryland, Baltimore, as well as a speaker for Nutricia North America/Danone, Nestle Health Science, and The Neurology Center in Rockville, Maryland. She receives royalties from Demos/Springer Publishing Company. Dr. Jane B. Allendorfer is funded by grants from NIH (R01HD102723 [PI]), the Evelyn F. McKnight Brain Institute, and LivaNova Inc.; has research previously funded by the US Department of Defense, State of Alabama, and the Charles L. Shor Foundation; has received honoraria from the Cleveland Clinic and the University of Auckland and travel funds from the International League Against Epilepsy; has served as a consultant for LivaNova Inc.; and serves as an editorial board member for Epilepsy & Behavior Reports. The remaining authors have no conflicts of interest relevant to this research activity.

## ETHICS STATEMENT

We confirm that we have read the Journal's position on issues involved in ethical publication and affirm that this report is consistent with those guidelines.

## Data Availability

The data that support the findings of this study are available on request from the corresponding author. The data are not publicly available due to privacy or ethical restrictions.
